# Effects of Dietary Selenium Nanoparticle Supplementation of a Low-Selenium Basal Diet on Nutrient Digestibility, Hemato-Biochemical Indices, Reproductive and Lactational Performance of Ossimi Sheep

**DOI:** 10.3390/ani16213469

**Published:** 2026-11-03

**Authors:** Emadeldien Mohamed Ibrahim, Mohamed Shehab-El-Deen, Ibrahim H. Algherair, A. A. AbdElhakeam, Yassin Kassim

**Affiliations:** 1 Animal and Poultry Production Department, Faculty of Agriculture, Minia University, El-Minya 61519, Egypt; profabdelhakeam@mu.edu.eg (A.A.A.); yassin.kassim@mu.edu.eg (Y.K.); 2 Department of Animal and Poultry Production, College of Agriculture and Food, Qassim University, Buraydah 51452, Saudi Arabia; m.shehabeldeen@qu.edu.sa; 3 Animal Production Department, Faculty of Agriculture, Suez Canal University, Ismailia 41522, Egypt; 4 Department of Animal and Fish Production, College of Agriculture and Food Sciences, King Faisal University, P.O. Box 402, Al-Ahsa 31982, Saudi Arabia

**Keywords:** nano-selenium, nutrient digestibility, ram semen quality, ewe fertility, total antioxidant capacity, triiodothyronine, milk composition, pre-weaning growth, trace mineral nutrition

## Abstract

Thirty Ossimi ewes and twelve Ossimi rams were used to evaluate the effects of supplementing a low-selenium (Se) basal diet with Se nanoparticles (Se-NP) on nutrient digestibility, hemato-biochemical indices, and reproductive-lactational performance. Ewes and rams were randomly allocated to two groups: a control group fed the basal diet (estimated Se content 0.005–0.014 mg/kg dry matter, DM) and a Se-NP group fed the same basal diet supplemented with 0.3 mg Se-NP/kg DM. Repeated measurements were analyzed with linear mixed models. In rams, dietary Se-NP increased (*p* < 0.001) ejaculate volume, sperm concentration, progressive motility, and serum testosterone levels compared with the control. In the Se-NP breeding group, the conception rate was numerically higher (93.3%) compared with 60.0% in the control group. Dietary Se-NP increased (*p* < 0.001) total antioxidant capacity, blood Se concentration, serum triiodothyronine (T_3_), the T_3_/thyroxine (T_4_) ratio, nutrient digestibility coefficients and nutritive values in both rams and ewes. Maternal Se-NP supplementation also increased (*p* < 0.001) milk yield, fat corrected milk, milk composition and daily yields of milk fat, protein, and lactose, as well as the birth weight, weaning weight and average daily gain of suckling lambs. Under the conditions of this study, Se-NP supplementation of a low-Se diet during the breeding period was associated with improved semen characteristics, lactational, and digestive performance; because ram and ewe supplementation were applied jointly within breeding groups and the sample size was limited, these findings should be confirmed in larger studies.

## Simple Summary

Reproductive efficiency and milk production are major determinants of profitability in sheep flocks. Selenium is an essential trace element involved in antioxidant defense, thyroid hormone metabolism, and reproduction, and selenium nanoparticles have been proposed as an efficient way of supplying it. In this study, Ossimi rams and ewes fed a low-selenium basal diet either received no supplement or were supplemented with selenium nanoparticles (0.3 mg/kg dry matter), starting three weeks before mating. Supplemented rams had higher ejaculate volume, sperm concentration, sperm motility, and blood testosterone, and numerically more ewes lambed in the supplemented breeding group. Supplemented ewes also showed higher nutrient digestibility, milk yield and milk component yields, and their lambs were heavier at birth and grew faster before weaning. Because rams and ewes in each breeding group received the same diet and the number of animals was limited, these results indicated that correcting a low dietary selenium supply with selenium nanoparticles is a promising strategy that should be confirmed in larger studies.

## 1. Introduction

Reproductive success in sheep production depends equally on the fertility of both ewes and rams. Although hormonal, nutritional, and genetic strategies have traditionally been used to enhance ewe fertility and prolificacy [1,2,3], the scientific literature underscores that ram-related factors—particularly semen characteristics—significantly affect flock reproductive outcomes [4,5]. From a practical standpoint, rams are pivotal as a single ram can mate numerous ewes, with ram:ewe ratios ranging from 1:25 to 1:100 depending on the production system [4,6,7]. Consequently, improving ram fertility can affect the reproductive, productive, and economic efficiency, ultimately shaping the flock’s sustainability [4,8]. However, multiple interacting factors influence ram fertility, including photoperiod, genetics, nutrition, and overall management practices [9,10]. Among these, nutritional status—particularly adequate trace mineral supplementation—is fundamental for maintaining optimal productive and reproductive function [4,11]. Furthermore, oxidative stress is increasingly recognized as an important factor that compromises male reproductive performance [12,13]. Overproduction of reactive oxygen species (ROS) can induce oxidative damage to spermatozoal lipids, proteins, and cellular DNA, impairing motility, cell viability, and overall fertilizing capacity [12,13,14]. Consequently, dietary interventions that bolster antioxidant defense mechanisms can enhance semen quality and optimize livestock reproductive outcomes [15,16].

Selenium (Se) is an essential trace element with a well-established role in male reproduction, primarily through its incorporation into selenoproteins such as glutathione peroxidase, which protect spermatozoa from oxidative damage [17]. Beyond its antioxidant functions, Se is required for testosterone biosynthesis and normal sperm development, and lower sperm Se concentrations have been associated with abnormal motility in rams [18,19,20]. In ewes, Se deficiency causes muscular dystrophy in lambs, contributes to fertility issues, abortions, and retained placenta, and impairs immunity and productivity in both dams and offspring [21,22]. At the molecular level, Se serves as a catalytic center for glutathione peroxidase [23], and mediates redox reactions within thioredoxin reductase [24], while acting as an essential cofactor for iodothyronine deiodinases that regulate thyroid hormone activation [14,25]. Given these multifaceted roles, adequate Se concentration in animal feed is critical for maintaining metabolic health, immune function, antioxidant defense, and reproductive performance [16].

Traditional Se supplementation in animal diets uses either inorganic forms (e.g., sodium selenite) or organic forms (e.g., selenium yeast), both of which have significant limitations [14,26,27,28]. Inorganic minerals are particularly susceptible to antagonisms in the digestive tract, leading to low absorption rates and necessitating higher doses that may contribute to environmental pollution. While organic sources improve on inorganic forms, they remain costly and still exhibit suboptimal bioavailability [14,26,27,28]. These limitations have driven the search for more advanced and effective mineral delivery systems.

Advances in nanotechnology have introduced Se nanoparticles (Se-NP), with particle diameters ranging from 1–100 nm, which have been proposed to offer advantages in animal nutrition because of their unique physicochemical properties, including small size, high stability, hydrophobicity, and large surface-area-to-volume ratio [29,30]. These features enable efficient dispersion in biological media, enhanced cellular uptake, greater mucosal tissue interaction, longer gastrointestinal residence time, and faster diffusion into cells than larger particles [30,31]. Se-NP have also been reported to show lower toxicity, higher bioavailability and greater tissue retention than conventional Se sources [32,33], and their supplementation has been associated with improved nutrient digestibility, immune response, growth performance, testicular function, and overall health in livestock species [14,30,34,35,36,37,38,39].

Most previous studies with Se-NP in small ruminants have evaluated either males [40,41] or females and their offspring [39,40,41,42,43] separately, and several compared Se-NP with other Se forms on basal diets that already supplied appreciable amounts of Se. In contrast, little is known about the response of the whole breeding unit -rams, ewes, and their suckling lambs- when Se-NP is used to correct a low-Se basal diet, as is common in intensive systems based on low-Se feedstuffs in Egypt. Information on the semen characteristics, reproductive outcomes, lactation, and pre-weaning growth in local breeds such as the Ossimi is particularly scarce. The present study did not compare Se-NP with other Se sources; rather, it compared Se-NP supplementation with an unsupplemented low-Se control.

We hypothesized that supplementing a low-Se basal diet with 0.3 mg Se-NP/kg DM, starting three weeks before mating, would improve antioxidant and thyroid status and nutrient digestibility and would thereby be associated with better semen characteristics in rams, better reproductive outcomes in the breeding groups, higher milk production in ewes, and faster pre-weaning growth of their lambs. Therefore, the objectives were to evaluate the effects of Se-NP supplementation of low-Se basal diet on nutrient utilization, hemato-biochemical profiles, antioxidant status, semen characteristics and reproductive and lactational performance of Ossimi rams and ewes, and on the growth of their suckling lambs.

## 2. Materials and Methods

The ethical committee for the care and use of animals in education and scientific research, Faculty of Agriculture, Minia University, El-Minya, Egypt, formally approved all experimental procedures involving animals under approval No. MU FA 0301123.

### 2.1. Animal Feeding and Management

This study was conducted at the Experimental Farm of the Animal Production Department, Faculty of Agriculture, Minia University, El-Minia, Egypt. The experimental animals consisted of thirty Ossimi ewes (43.3 ± 2.27 kg) and twelve Ossimi rams (49.54 ± 2.15 kg), all one year of age at the start of the trial.

The animals were housed in well-ventilated, semi-open stables, with rams and ewes of each treatment kept in separate group feeding pens except during the daily mating periods. Prior to the commencement of the experimental supplementation, all animals underwent a 21-day adaptation period to acclimatize to the experimental housing facility, group-pen environment, and management routines while being fed the unsupplemented basal diet. Following this adaptation phase, in a completely randomized design, ewes and rams with similar initial body weights were randomly allocated to two equal, independent groups (6 rams and 15 ewes per group). The control group (CON) received a basal diet (Table 1) to meet their nutrient requirements based on their live body weight and physiological status [44]. The treatment group received the same basal diet supplemented with 0.3 mg Se-NP/kg DM. This value refers to supplemental Se provided as Se-NP; therefore, the total dietary Se concentrations was approximately 0.314 and 0.305 mg/kg DM in the supplemented diets of rams and ewes, respectively, compared with 0.014 and 0.005 mg/kg DM in the control diets (Table 1). The dose was chosen to bring total dietary Se to the upper end of the range recommended for sheep (0.1–0.3 mg/kg DM) [44]. The Se-NP product was obtained from Sigma-Aldrich (St. Louis, MO, USA); according to the manufacturer’s specifications, it consisted of elemental Se with a particle size <80 nm and 99.9% purity. No additional characterization (e.g., morphology, particle size distribution, agglomeration state, surface properties, or stability after incorporation into the diet) was performed by the authors. The Se-NP was initially pre-mixed with finely ground yellow corn before being incorporated into the remaining basal diet ingredients. The diets were offered as a non-pelleted total mixed ration (mash).

Dietary supplementation for rams began three weeks pre-mating and continued throughout the breeding phase and the subsequent digestibility trial. However, the ewes were treated starting three weeks before mating, with supplementation continuing throughout mating, gestation, and suckling, as well as the subsequent digestibility trial. All animals were offered their respective feed twice daily at 08:00 and 18:00 h, with unrestricted access to fresh drinking water. Supplementation was started three weeks before mating to cover the period of final follicular development preceding mating in ewes, as applied in short-term pre-mating nutritional programs [3].

We recorded individual ram body weights at the start of the study and throughout the breeding period. The ewes’ body weights were documented at the start of the experiment, on lambing day, and monthly throughout the suckling period. The birth weights of the resulting lambs were recorded within 24 h of parturition, then monitored weight biweekly during the suckling period to calculate average daily weight gain. The mean dry matter intake (DMI) was determined during the final week of each month: The feed offered and the feed refused were weighed daily and DMI was calculated as DM offered minus DM refused per pen and divided by the number of animals.

During the breeding phase, every day, three of the six rams of the same treatment group were selected at random and joined with the ewes for 3 h outside the feeding times, so that all six rams of each group were used in daily rotation, over a total duration of 36 days. Key reproductive parameters, including the number of ewes exposed to rams, ewes lambing, total lambs born, twin-bearing ewes, and stillborn lambs, were recorded for each group. Maternal reproductive efficiency and offspring traits—specifically conception rate, barrenness rate, fecundity, prolificacy, live-birth rate, as well as lamb mortality and survival rates up to day 7 of age—were subsequently calculated as follows: conception rate (%) = ewes lambing/ewes exposed × 100; barrenness rate (%) = ewes not lambing/ewes exposed × 100; fecundity (%) = lambs born/ewes exposed × 100; prolificacy (litter size, %) = lambs born/ewes lambing × 100; live-birth rate (%) = lambs born alive/lambs born × 100; stillbirth rate (%) = stillborn lambs/lambs born × 100; lamb mortality up to day 7 (%) = lambs dead (including stillbirths) by day 7/lambs born × 100; and lamb survival up to day 7 (%) = lambs alive on day 7/lambs born × 100. Because prolificacy expresses the number of lambs per ewe lambing, values above 100% indicate multiple births. Because rams and ewes within each breeding group received the same diet and were mated as a group, the reproductive outcomes reflect the combined effect of paternal and maternal supplementation; the individual ewe was used as the observational unit for binary reproductive outcomes, and the separate contributions of ram and ewe supplementation cannot be distinguished.

### 2.2. Dietary Sampling and Laboratory Analysis

Representative feed samples were collected daily during the final week of each month throughout the experimental period to perform a composite sample. One aliquot of the composite sample was dried at 105 °C in a forced-air oven until a constant weight was achieved for dry matter (DM) determination. The remaining aliquot was dried at 70 °C to a constant weight, finely ground, and stored in hermetically sealed jars for subsequent laboratory analysis. The feed samples were analyzed for DM, organic matter (OM), crude protein (CP), crude fiber (CF), ether extract (EE), and ash content according to AOAC protocols [45]. Neutral detergent fiber (NDF) and acid detergent fiber (ADF) were determined using established methodologies [46]. Grab fecal samples were collected from the rectum of each animal twice daily at 07:00 and 14:00 h before feeding during the final week of the last month. These fecal samples were pooled, dried at 70 °C until a constant weight was achieved, and analyzed for DM, OM, CP, CF, NDF, ADF, EE, and ash. Total tract digestibility coefficients for DM, OM, CP, CF, NDF, ADF, and EE were calculated for each animal using acid insoluble-ash (AIA) as an internal marker [47]. The AIA concentrations of feed and fecal samples were determined analytically: samples were ashed, the ash was boiled in 2 N HCL, filtered through ashless filter paper, washed free of acid with hot distilled water, and re-ashed, and the residue was weighed as AIA [47]. Apparent DM digestibility (%) was calculated as 100 − [100 × (AIA in feed/AIA in feces), and that of each nutrient as 100 − [100 × (AIA in feed/AIA in feces) × (nutrient in feces/nutrient in feed)]. Digestible crude protein (DCP) was calculated as CP × CP digestibility, and total digestible nutrients (TDN) as digestible CP + digestible CF + digestible NFE + 2.25 × digestible EE. TDN and DCP were used because they are the units of the feeding standards applied in Egypt and in previous studies with Ossimi sheep [39,42]; for comparison with metabolizable energy (ME) based systems, ME was estimated from TDN as ME (Mcal/kg DM) = 0.82 × (0.04409 × TDN, %). Table 1 presents the approximate analysis of the total mixed ration (TMR).

### 2.3. Scrotal Circumference and Semen Physical Characteristics

The scrotal circumference (SC, cm) of the experimental rams was measured prior to morning feeding at the beginning of the trial and then at biweekly intervals using a scrotal measuring tape (Nasco International, Inc., Atkinson, WI, USA) and the SC average for each animal was recorded at 0, 30, 60 and 90 days. For semen collection, rams were sexually stimulated by allowing two false mounts during a 2–3 min restraint period immediately preceding ejaculation [48]. Ejaculates were collected weekly between 08:00 and 10:00 a.m. using an artificial vagina, for six consecutive weeks during the supplementation period. This manual collection approach was intentionally selected over electroejaculation, as the latter technique has been documented to alter seminal characteristics and compromise sperm quality, thereby establishing the artificial vagina as the preferred tool for objective fertility assessments [49,50]. At the time of collection, the inner liner and rubber sleeve temperature of the artificial vagina was maintained between 41 °C and 43 °C. A sanitized, dry inner liner and a graduated plastic collection tube were used for each individual ejaculate, with lubrication achieved using sterile white petroleum jelly applied via a sterile glass rod.

At each weekly collection session, two successive ejaculates were obtained separately for subsequent laboratory assessment. Semen samples were immediately transferred to the laboratory for processing upon collection. Ejaculate volume (EV, ml) was quantified directly to the nearest 0.1 mL using the graduated markings on the transparent plastic collecting tubes. Sperm progressive motility (SM, %) was evaluated by placing a drop of semen on a warm-stage research microscope (OPTIKA, B-383FL, Ponteranica, Italy) adjusted to 37 °C, coupled to a closed-circuit TV camera and a color monitor for real-time visualization. Sperm cell concentration (Sperm C, ×10^6^/mL) was determined using the standard hemocytometer technique.

### 2.4. Milk Sampling and Analysis

Daily milk yield was measured weekly from each ewe from the fifth day post-lambing until weaning, utilizing the classic lamb suckling technique [51]. Lambs were separated from their dams at 16:00 h on the day preceding the measurement. On the following test day, lambs were weighed at 08:00 h, allowed to suckle until satisfied, reweighed, and isolated once more. We hand-milked any residual milk remaining in the udder and weighed it. At 16:00 h, the lambs were reweighed before and after a second suckling session, and any subsequent residual milk was again hand-milked and weighed. The net milk consumption by each lamb during the morning and afternoon intervals was calculated from the weight differentials before and after suckling. The total hand-milked volume (morning plus afternoon) was added to the suckling intake to estimate the total 24 h milk production. Because the lambs were separated at 16:00 h on the previous day and the test-day sucklings took place at 08:00 h and 16:00 h, the two intervals (16 h and 8 h) covered a standardized 24 h period for all ewes. For ewes rearing twins, the intakes of both lambs were summed. Lamb sex and litter size were recorded but were not included as covariates because there were only two twin litters (both in the Se-NP group). We collected representative milk samples individually each week after parturition until weaning. These samples were analyzed for protein, fat, lactose, solid not fat (SNF), and ash percentage via infrared spectrophotometry using a MilkoScan unit (Model 130 series, Type 10900, FOSS Electric, Hillerød, Denmark). Fat-corrected milk yield (6% FCM for sheep) was calculated using the following standard equation: FCM (kg) = daily milk yield (kg) × (0.428 + [0.095 × fat %]) [52].

### 2.5. Hematological Profiling and Leukocyte Differentiation

Heparinized blood samples were collected via jugular venipuncture from all the experimental animals at monthly intervals. All sampling was performed at 08:00 h before morning feeding or drinking. Immediately following collection, the heparinized whole blood samples were analyzed to determine hemoglobin (Hb) concentration, packed cell volume (PCV), red blood cell counts (RBCs), and white blood cell counts (WBCs). Additionally, we quantified differential leukocyte percentages, including lymphocytes, neutrophils, monocytes, basophils, and eosinophils. These hematological profiles were evaluated utilizing an automated veterinary hematology analyzer (VetScan HM5 Hematology System, Abaxis Uk Ltd, York, North Yorkshire, UK).

### 2.6. Serum Biochemical and Hormonal Assays

Similarly, non-heparinized blood samples were obtained concurrently under the same sampling conditions to harvest serum. The samples were allowed to clot and subsequently centrifuged at 3000 rpm for 10 min to obtain serum. The harvested serum aliquots were stored in a freezer maintained at −20 °C until subsequent biochemical profiling. Serum concentrations of triiodothyronine (T_3_), thyroxine (T_4_), testosterone (rams), and progesterone (ewes, at mating, during pregnancy and at weaning) were quantified via the enzyme-linked immunosorbent assay (ELISA) technique using commercial EIA kits (Prechek Bio, Inc., Atlaslink Technology, California, USA). The T_3_/T_4_ ratio was subsequently calculated. Serum total antioxidant capacity (TAC) was analyzed colorimetrically using a spectrophotometer (STAT-LAB SZSL60-SPECTRUM, Spectrum Diagnostics, Bangalore, India) with specialized diagnostic kits (Bio-diagnostic Company, Dokki, Giza, Egypt). Serum Se concentrations at the initial and final stages of the experimental period were determined by electrothermal atomic absorption spectrometry (PerkinElmer Model 5100, PerkinElmer, Springfield, IL, USA). The specific biochemical and elemental profiles were conducted at the laboratories of the Egyptian Atomic Energy Authority in Cairo, Egypt.

### 2.7. Statistical Analysis

All analyses were performed in R (version 4.3.3; R Foundation for Statistical Computing, Vienna, Austria) using the packages nlme, lme4, lmerTest, and emmeans. The individual animal was the experimental unit for all individually measured variables. Normality of residuals was examined with residual plots and the Shapiro–Wilk test. Variables measured once per animal (IBW, FBW, nutrient digestibility coefficients, and nutritive values) were compared between groups using one-way ANOVA (equivalent to the two-tailed unpaired Student’s *t*-test for two groups). Variables measured repeatedly on the same animals were analyzed with linear mixed models (generalized least squares with a structured residual covariance matrix for the repeated measurements on each animal), including treatment, sampling time, and the treatment × time interaction as fixed effects.

In rams, these variables were hematological and serum biochemical variables (months 1, 2, and 3), serum testosterone and SC (days 30, 60, and 90), and semen characteristics (months 1, 2, and 3; weekly collections and both ejaculates averaged per ram and month; in ewes, they were hematological and serum biochemical variables and serum progesterone (mating, pregnancy, and weaning) and milk yield and composition (months 0 to 3 of lactation; weekly test-day records averaged per ewe and month). For all variables with a pre-treatment measurement (day 0 or start of the trial), this value was included as a covariate, and the pre-treatment values of the two groups were compared by one-way ANOVA. The covariance structure (compound symmetry, first-order autoregressive, or unstructured) was selected for each variable by Akaike’s information criterion, and denominator degrees of freedom were obtained with the between-within method. Least-squares means of the two groups were compared at each sampling time, with Sidak adjustment for the number of sampling times. Serum Se at the end of the trial was analyzed by analysis of covariance with the day-0 concentration as covariate.

In ewes, blood samples during pregnancy and at weaning were analyzed for pregnant and lactating ewes only; the mixed model uses all available observations without imputation. DMI was recorded per pen; because the pen was the experimental unit for intake, DMI, DCPI, and TDNI are presented descriptively and were not tested statistically. Lamb birth weight, weaning weight, and ADG were analyzed with a linear mixed model including maternal treatment and birth type (single or twin) as fixed effects and the dam as a random effect, to account for twins sharing the same dam, with Kenward-Roger degrees of freedom; lamb sex was not available in the data. Binary reproductive and offspring survival outcomes (conception, barrenness, twin-bearing, live birth, stillbirth, mortality, and survival) were compared using the two-tailed Fisher’s exact test, with the ewe or the lamb as the observational unit, and the difference between groups is also reported as a risk difference with its 95% confidence interval (Newcombe hybrid score method). Because rams and ewes were supplemented jointly and mated as one breeding group per treatment, these outcomes reflect the combined effect of paternal and maternal supplementation. Fecundity and prolificacy are lamb-to-ewe ratios and are presented descriptively. Results are presented as least-squares means and pooled SEM. Statistical significance was set at *p* < 0.05.

## 3. Results

### 3.1. Effects of Se-NP Supplementation on Rams’ Productive Performance

#### 3.1.1. Body Weight and Feed Intake

The IBW did not differ between groups, and dietary Se-NP did not affect the FBW of Ossimi rams (*p* > 0.05; Table 2). The DCPI and TDNI were numerically higher in the Se-NP group (163.3 and 1040 g/head/day, respectively) than in the control group (147.6 and 980.3 g/head/day).

#### 3.1.2. Nutrient Digestibility and Feeding Values

Dietary supplementation with Se-NP increased the total tract digestibility of nutrients and overall nutritive values in Ossimi rams (Table 3). In particular, the Se-NP supplemented group showed higher (*p* < 0.001) in the digestibility coefficients of DM, OM, CP, and EE relative to the control group. The CF digestibility was also higher (*p* < 0.01) in the Se-NP group. Furthermore, the digestibility values of ADF (*p* = 0.017) and NDF (*p* = 0.043) exhibited significant improvements (*p* < 0.05) compared to the control group. Overall, dietary Se-NP inclusion significantly increased (*p* < 0.001) the DCP and TDN percentages compared with the control group.

#### 3.1.3. Hematological Parameters and Leukocyte Profiles

Hematological variables of the rams are presented in Table 4. Se-NP did not affect WBC count, PCV, or the percentages of neutrophils, basophils, and lymphocytes (*p* > 0.05). The Hb concentration (13.28 vs. 12.67 g/dL; *p* = 0.013) and the percentages of eosinophils (*p* < 0.001) and monocytes (*p* = 0.007) were higher in the Se-NP group, although the differences in leukocyte percentages were small in absolute terms. RBC count was lower in the Se-NP group (*p* = 0.007). Treatment × time interactions were detected for lymphocytes and monocytes (*p* < 0.001 for both): the monocyte percentage was higher in the Se-NP group in months 1 and 2 (*p* = 0.004 and *p* < 0.001) but not in month 3, and the lymphocyte percentage was lower in the Se-NP group in month 2 only (*p* = 0.028).

#### 3.1.4. Serum Hormonal Profiles, Selenium Concentrations, and Antioxidant Status

Serum hormonal profiles, Se concentrations, and antioxidant status of the rams are presented in Table 5. The Se-NP increased serum T_3_ (*p* < 0.001) and the T_3_/T_4_ ratio (*p* < 0.001), decreased serum T_4_ (*p* < 0.001), and increased serum TAC (*p* < 0.001). For T_3_, T_4_, and the T_3_/T_4_ ratio, the treatment × time interactions (*p* < 0.001) reflected differences that were confined mainly to month 3, whereas TAC was higher in the Se-NP group from month 1 onward. The serum Se did not differ between groups on day 0 (*p* = 0.473) but was higher in the Se-NP group at the end of the trial (0.556 vs. 0.286 μg/mL; *p* < 0.001). Serum testosterone did not differ on day 0 (*p* = 0.893); thereafter, the effects of treatment, time, and their interaction were significant (all *p* < 0.001), and testosterone was higher in the Se-NP group on days 30, 60, and 90 (Sidak-adjusted *p* < 0.001 at each time), with the difference increasing from 1.79 ng/mL on day 30 to 6.81 ng/mL on day 90.

#### 3.1.5. Scrotal Circumference and Semen Physical Characteristics

The SC and semen characteristics are presented in Table 6. The SC was higher in the Se-NP group (30.71 vs. 29.19 cm; *p* = 0.027), with a treatment × time interaction (*p* = 0.004); the time-specific difference was significant on day 60 (*p* = 0.029) but not on day 30 or day 90 (*p* = 0.508 and *p* = 0.056, respectively). Se-NP increased EV (*p* < 0.001), Sperm C (*p* < 0.001), and SM (*p* < 0.001); for EV and Sperm C, the treatment effect was consistent across months (treatment × time, *p* = 0.183 and *p* = 0.522), whereas for SM, the interaction (*p* = 0.006) reflected a larger difference in months 1 and 3.

### 3.2. Effects of Se-NP Supplementation on Ewes’ Productive and Reproductive Performance

#### 3.2.1. Body Weight and Feed Intake

Body weight and feed intake of Ossimi ewes are presented in Table 7. The IBW did not differ between groups, and Se-NP did not affect FBW (*p* > 0.05). The DMI, DCPI, and TDNI, recorded per pen, are presented descriptively; DCPI and TDNI were numerically higher in the Se-NP group.

#### 3.2.2. Digestibility and Feeding Values

Table 8 presents the nutrient digestibility coefficients and nutritive values of the experimental rations for ewes. Dietary Se-NP inclusion significantly increased (*p* < 0.01) the digestibility coefficients of DM, OM, CP, CF, and NDF, as well as the nutritive values of DCP and TDN in the treatment group compared to the CON group. Furthermore, Se-NP significantly improved EE digestibility (*p* < 0.05) in ewes. However, dietary treatments did not affect ADF digestibility (*p* = 0.6887).

#### 3.2.3. Hematological Parameters and Leukocyte Profiles

Hematological variables of the ewes are presented in Table 9. The Se-NP increased PCV (*p* < 0.001) and Hb concentration (*p* < 0.001), and both were higher in the Se-NP group at mating, during pregnancy, and at weaning (Sidak-adjusted *p* < 0.001 at each time). The RBC count was also higher in the Se-NP group (*p* = 0.002), but the treatment × time interaction (*p* < 0.001) showed that the difference was confined to weaning (*p* < 0.001). WBC count (*p* = 0.206) and the percentages of neutrophils, eosinophils, basophils, lymphocytes, and monocytes (*p* ≥ 0.321) were not affected by Se-NP. The eosinophil, basophil, and monocyte percentages already differed slightly between groups at the start of the trial (*p* < 0.001, *p* = 0.009, and *p* = 0.003, respectively), and these differences did not change during the trial.

#### 3.2.4. Serum Hormonal Profiles, Selenium Concentrations, and Antioxidant Status

Serum hormonal profiles, blood Se concentrations, and antioxidant status of the ewes are presented in Table 10. Se-NP increased serum T_3_ (*p* < 0.001), the T_3_/T_4_ ratio (*p* < 0.001), and serum TAC (*p* < 0.001), and these differences were present at mating, during pregnancy, and at weaning (Sidak-adjusted *p* < 0.001 at each time). Serum T_4_ was lower in the Se-NP group (*p* < 0.008), but only at weaning (*p* = 0.002; treatment × time, *p* < 0.001). Serum Se did not differ between groups at the start of the trial (*p* = 0.485) but was higher in the Se-NP group at its end (0.585 vs. 0.312 μg/mL; *p* < 0.001). Serum progesterone did not differ between groups at the start of the trial (*p* = 0.877). Thereafter, the effects of treatment, stage, and their interaction were significant (all *p* < 0.001): progesterone was higher in the Se-NP group during pregnancy (8.41 vs. 7.56 ng/mL; *p* < 0.001), whereas it was lower in the Se-NP group at weaning (2.13 vs. 4.77 ng/mL; *p* < 0.001); the difference at mating, although significant (*p* = 0.012), was negligible in magnitude (5.22 vs. 5.28 ng/mL). Pregnancy and weaning values refer to pregnant and lactating ewes, respectively.

#### 3.2.5. Reproductive Performance

In each group, 15 of the ewes exposed to rams, 14 lambed in the Se-NP group and 9 in the CON group, corresponding to conception rates of 93.33% and 60.00%, respectively (risk difference 33.3 percentage points, 95% CI 2.6 to 58.2; two-tailed Fisher’s exact test, *p* = 0.080); the barrenness rate was correspondingly lower in the Se-NP group (6.67% vs. 40.00%; Table 11). The 16 lambs born in the Se-NP group resulted from 12 single and 2 twin births, whereas all 9 lambs in the CON group were singles. Fecundity was 106.67% in the Se-NP group and 60.00% in the CON group, and prolificacy was 114.29% and 100.00%, respectively. The number of twin-bearing ewes, live-birth rate, stillbirth rate, lamb mortality up to day 7, and lamb survival up to day 7 did not differ between groups (*p* > 0.05). Given the small number of ewes per group and the group-mating design, these reproductive differences should be interpreted with caution.

#### 3.2.6. Milk Yield and Composition

Milk yield and composition of the ewes are presented in Table 12. Se-NP increased the concentrations of TS, fat, protein, lactose, SNF, and ash (*p* ≤ 0.028), as well as MY (675.0 vs. 517.3 g/day), FCM, and the daily yields of fat, protein, and lactose (all *p* < 0.001). For the composition variables, the treatment × time interactions (*p* < 0.001) showed that the differences were largest in month 3 of lactation; protein, SNF, and TS did not differ, or were slightly lower in the Se-NP group, in month 2. MY, FCM, and component yields were higher in the Se-NP group in every month.

#### 3.2.7. Suckling Lamb Growth Performance

The growth traits of suckling lambs are summarized in Table 13. In the mixed model including birth type as a fixed effect and the dam as a random effect, lambs of Se-NP-supplemented ewes had a higher birth weight (*p* < 0.001; birth type, *p* < 0.001), weaning weight (*p* < 0.001), and ADG (*p* < 0.001) than lambs of CON ewes. The observed weaning weight and ADG were 14.2% and 14.9% higher, respectively, in the Se-NP group.

## 4. Discussion

Regarding body weight parameters and feed consumption, the lack of significant differences in FBW and DMI in both rams and ewes between the experimental groups, coupled with the observed higher DCP and TDN intake values in the Se-NP group, aligns with previous literature reporting no adverse effects of Se-NP on DMI in dairy cows [53,54]. In line with these findings, Se-NP supplementation did not alter FBW, or DMI in Ossimi or Awassi ewes [39,42,55]. Taken together, the present results may imply that incorporating Se-NP into sheep rations at a dose of 0.3 mg/kg DM does not adversely affect dietary palatability or subsequent DMI, which is consistent with reports that Se-NP supplementation is unlikely to alter feed intake in ruminant species [43].

With respect to nutrient digestibility and nutritive values, the improvements observed in both rams and ewes supplemented with Se-NP are consistent with previous investigations in Barki ewes and dairy cows [43,56], in which Se supplementation increased the digestibility of fiber fractions, possibly by stimulating enzymatic activity and modulating rumen microbial populations [57]. One possible explanation is that the antioxidant activity of Se-NP mitigates ruminal oxidative stress, providing a more favorable environment for fermentation and for cellulolytic bacteria and continuous Se supplementation has been associated with greater cellulolytic bacterial populations and fiber degradation in the rumen [57,58]. However, rumen fermentation and microbial populations were not measured in the present study, so these mechanisms remain speculative and should be verified in future Se-NP trials. In sheep, elemental Se-NP increased digestibility and rumen fermentation compared with sodium selenite [59]; Se-NP consists of elemental Se and is therefore distinct from both conventional inorganic salts (e.g., sodium selenite) and organic forms (e.g., selenomethionine or Se-yeast). Recent studies in sheep also reported higher nutrient digestibility coefficients and nutritive values with Se-NP at 0.3 mg/kg DM than with unsupplemented basal diets [39,42,60], and Se supplementation may improve protein utilization and ruminal fermentation [27,59]. The physicochemical properties of nanoparticles, such as a high surface-area-to-volume ratio and small particle size, have been proposed to favor gastrointestinal absorption [31,61], although absorption and bioavailability were not measured here.

In terms of hematological dynamics, the previous literature evaluating dietary Se-NP supplementation in small ruminants remains limited and discordant. For instance, dietary Se inclusion did not modify baseline erythroid profiles, including RBCs, hematocrit values, and hemoglobin concentrations in either goats or calves [62,63]. In the current study, dietary Se-NP supplementation increased RBC count, PCV, and Hb concentration in Ossimi ewes and Hb concentration in Ossimi rams, whereas PCV in rams was not affected and RBC count was lower in the Se-NP rams, a difference that was already present before supplementation. Biochemically, Se preserves the structural stability and oxidative resistance of erythrocyte membranes, whereas its systemic deficiency remains a documented factor inducing hemolytic anomalies and anemia [64]. Concerning the leukocyte defense network, Se supplementation has been reported to increase WBCs in ruminants [65,66,67]. In this context, Se serves as a key micronutrient that supports cellular immune competence and leukocyte viability by shielding immune cells from peroxidative injury during metabolic demand [68]. This protective role may explain the differences observed in specific leukocyte differential counts in the present study, namely higher eosinophil and monocyte percentages in rams; these differences were, however, small in magnitude and of uncertain biological relevance. Conversely, earlier research indicated that parenteral Se administration did not modify leukocytic profiles in alternative ruminants [62,66]. This discrepancy across the literature may be attributed to variations in animal species, sex, age, physiological status, and the specific dietary Se dosage and supplemental chemical form.

Serum biochemical profiles serve as sensitive and rapid indicators of nutritional status and animal health in ruminant production systems. The circulating indices measured in the present study remained within reference ranges for sheep [69]. In terms of dietary and systemic Se status, the baseline Se concentrations calculated based on NRC tables within the basal diets of both rams and ewes (0.014 and 0.005 mg/kg DM, respectively) were well below the dietary requirement for sheep. The standard dietary requirement for Se typically ranges from approximately 0.1 to 0.3 ppm on a DM basis in ruminants [44,70]. Following intestinal absorption, Se enters the blood or is transferred into milk, making circulating profiles a reliable proxy for evaluating bio-utilization and tissue distribution in small ruminants [54]. As a key bio-indicator in small ruminants, serum Se concentrations spanning between 0.005 and 0.360 μg Se/ml have been defined as clinically adequate within the literature [71]. Comparable responses in serum Se were reported in Barki ewes supplemented with Se-NP [43]. Importantly, although the calculated Se content of the basal diet was very low, serum Se concentrations of the CON animals (approximately 0.18–0.31 μg/mL; Table 5 and Table 10) were within the range considered adequate for sheep [71] and increased during the trial without supplementation. This indicates that the actual Se intake of the control animals was higher than that calculated from tabulated feed values (e.g., because of regional variation in the Se content of feedstuffs or Se in drinking water) and that the control animals were not clinically Se-deficient. In contrast, the final serum Se concentrations of the Se-NP group (approximately 0.56–0.59 μg/mL) exceeded the upper limit of the adequate range cited above [71], which reinforces the need for a direct evaluation of Se retention and safety. Furthermore, the dietary Ca and vitamin E contents, which interact with Se requirements and with the expression of Se deficiency, were not determined, and the basal Se concentration was calculated rather than analyzed; the present results should therefore be considered tentative with respect to Se requirements. Because Se-NP may be absorbed more readily than some other Se forms, the present data may also be useful for re-evaluating Se recommendations for breeding sheep fed Se-NP.

Concurrently, serum TAC levels were higher in the Se-NP-supplemented animals than in the control group. It has been proposed that the large surface area of Se nanoparticles provides active sites that scavenge and neutralize ROS [31]. These findings align with earlier reports indicating that supplemental Se drives antioxidant enzyme kinetics and maximizes TAC concentrations in small ruminants [60,72]. In this context, systemic Se availability is known to support the synthesis and activity of selenoproteins, notably glutathione peroxidase (GPx)and thioredoxin reductase, which collectively neutralize lipid peroxides and preserve cellular membrane integrity against peroxidative damage during high metabolic demand [68]; however, GPx and thioredoxin reductase activities were not measured in the present study.

Regarding endocrine regulation, dietary Se-NP inclusion was associated with higher serum T_3_ concentrations and T_3_/T_4_ ratios in both rams and ewes. Earlier investigations also showed that Se-NP supplementation enhances circulating thyroid hormone profiles in small ruminants [42,73]. This response is consistent with the higher blood Se concentrations, because circulating Se is an obligate structural component of selenocysteine, which forms the catalytic active site of thioredoxin reductases and iodothyronine deiodinases [14,25,74]. Specifically, Se availability influences the activity of 5′-deiodinase, a selenoprotein enzyme responsible for the peripheral monodeiodination of T_4_ into the metabolically active T_3_ hormone—a metabolic process highly sensitive to systemic Se status that orchestrates cellular metabolism, tissue growth, and overall productive performance [68]. Consistently, serum T_4_ was lower in the Se-NP rams and, at weaning, in the Se-NP ewes.

In male reproduction, Se is preferentially retained in the testis [75], where it supports testosterone biosynthesis and the maturation of spermatozoa [76]. Furthermore, Se serves as an obligate structural component of a specialized class of selenoproteins, including glutathione peroxidase (GPx) isoforms and other critical functional proteins localized within the testis, epididymis, and mature sperm architecture [77,78]. For instance, GPx1 and GPx3 are selectively expressed within the epididymal epithelia, functioning as a primary biochemical shield that protects the epididymal parenchyma and maturing spermatozoa from oxidative stress. Crucially, the phospholipid hydroperoxide GPx isoform (GPx4) acts as a vital antioxidant enzymatic framework safeguarding developing spermatids against peroxidative DNA damage. Beyond its catalytic role, GPx4 is a major structural constituent of the sperm mitochondrial capsule within the midpiece, thereby governing the structural stability, axonemal integrity, and forward motility kinetics of spermatozoa [68,79].

The increase in ejaculate volume observed with Se-NP supplementation may be related to the role of Se in germ cell proliferation and differentiation [80] and in the development and secretory activity of the accessory sex glands reported in other studies [81,82]. The higher sperm concentration is consistent with this interpretation and with the relationships between Se status and quantitative semen characteristics described previously [19,83]. Selenoproteins have also been implicated in chromatin condensation during spermatogenesis, sperm maturation, and morphology [84], and Se has been shown to support Sertoli cell function and to protect Leydig cells and the germinal epithelium from oxidative damage [19,68]. However, testicular histology, germ-cell proliferation, Sertoli- and Leydig-cell function, sperm chromatin, and GPx activity were not evaluated in the present study; these mechanisms, therefore, remain hypotheses that require direct testing.

Regarding spermatozoal kinetic traits, ROS serve an essential role in fundamental sperm functions, including fertilization, capacitation, acrosomal reaction, hyperactivity, and motility [85]. However, elevated ROS levels harm sperm quality through membrane damage and reduced motility, partly by impairing ATP utilization by the flagellar contractile apparatus [86,87]. Se supplementation may counteract oxidative stress by improving the antioxidant status, thereby limiting sperm damage, improving motility, and reducing morphological abnormalities [31]. Additionally, the insulin-like properties of Se may further enhance progressive motility by stimulating glucose transport and increasing both aerobic and anaerobic glycolysis rates [88,89,90]. Sperm morphology was not assessed in the present study; however, improved motility has been associated with a lower incidence of structural defects in other studies. For instance, earlier reports indicated that Se supplementation reduced the number of sperm with broken flagella [91]. In contrast, others observed that Se decreased mitochondrial abnormalities in the sperm tail midpiece [41]—both of which may contribute to the overall enhancement of sperm motility. Furthermore, the administration of Se-NP on ram or buck feed increases semen motility and intact membrane cells and reduces declined acrosome integrity compared with control [40], an effect attributed to the antioxidant activity of Se-NP and GPx, which may protect the sperm plasma membrane and counter peroxidative decay during spermatogenesis [68].

Interstitial Leydig cells biosynthesize testosterone via steroidogenesis, a complex enzymatic pathway inherently susceptible to peroxidative disruption by ROS. In these cells, ROS are generated primarily via the mitochondrial electron transport chain and cytochrome P450 enzymatic reactions in both mitochondria and microsomes [92]. When the endogenous antioxidant defense machinery is compromised or insufficient, steroidogenic function declines, culminating in decreased androgen output [93]. Conversely, sustained antioxidant administration restores and enhances circulating testosterone concentrations [94]. Selenium, in particular, acts as a potent redox regulator that safeguards testicular architecture and cellular homeostasis [95]. This framework could explain the higher serum testosterone concentrations observed in the Se-NP supplemented rams, which may be related to the protection of Leydig cells and the maintenance of the intracellular redox balance required for steroidogenesis [19,68]; however, Leydig-cell integrity and testicular redox status were not measured in the present study. These outcomes align with earlier research showing that dietary inclusion of Se-NP at 0.3 mg/kg DM effectively elevates serum testosterone profiles in small ruminants relative to control groups [96].

Compelling evidence from earlier investigations on rams showed that the co-administration of Se and vitamin E optimizes ejaculate volume [97]. Similarly, studies on Se efficacy in small ruminants showed improvements in libido scores, higher ejaculate volumes, accelerated spermatozoal motility, and higher sperm cell concentrations [98]. Consistent with the quantitative and qualitative improvements reported in the current study, findings in bucks indicate that dietary Se supplementation effectively increases semen volume, overall spermatozoal traits, and systemic testosterone concentrations [83,99,100]. Physiologically, these synchronized improvements may stem from Se’s role in driving spermatogenesis and supporting the enzymatic machinery required for systemic testosterone synthesis, both vital biocatalytic components that govern reproductive longevity and fertility in male animals [19,68,101].

Flock fertility, defined as the proportion of ewes lambing relative to those exposed to rams during the breeding season, constitutes a pivotal economic trait governing the overall production efficiency and subsequent profitability of sheep production systems [10]. Systemic deficiencies in essential micronutrients, particularly Se, copper, cobalt, manganese, iodine, iron, and zinc, are well documented to precipitate reproductive failure in ruminants, with Se deprivation directly reducing fertilization rates and increasing embryonic mortality [11,34]. Furthermore, a marginal lack of Se impairs systemic reproductive dynamics, resulting in silent heat, cystic ovarian configurations, and the delivery of unthrifty or unhealthy offspring [102]. In the current study, the Se-NP breeding group had a numerically higher conception rate (93.3% vs. 60.0%; risk difference 33.3 percentage points, 95% CI 2.6 to 58.2), although the two-tailed Fisher’s exact test did not reach significance (*p* = 0.080) with 15 ewes per group; this is in agreement with a previous report of improved reproductive performance in ewes and Zaraibi goats receiving Se-NP [103,104]. These maternal systemic improvements are further supported by earlier investigations noting that strategic Se administration before mating or during late gestation significantly increases pregnancy rates, lambing percentages, and twinning frequencies in small ruminants [81,105]. Two aspects must, however, be considered when interpreting these results. First, the conception rate (60%) and the early lamb losses in the CON group were poor compared with those expected in well-managed flocks. The calculated Se content of the basal diet suggested a low Se supply; however, blood Se concentrations of the control ewes remained within the adequate range, so frank Se deficiency is unlikely to be the only explanation. Other factors probably contributed, including the age of the ewes, the restricted mating schedule (3 h/day for 36 days), and the limited daily exposure to rams (three rams for 3 h/day). The reproductive data of the control group may therefore not be representative of normally managed adult flocks, and the magnitude of the difference between groups should be interpreted with caution. Second, because rams and ewes in each breeding group received the same diet and were mated as a group (three randomly selected rams of the same group per day, rotated among the six rams) and paternity was not determined, the difference in conception cannot be attributed specifically to ewe supplementation, ram supplementation, the improved semen characteristics, or their interaction. Studies with reciprocal (ram × ewe) treatment combinations or artificial insemination with semen from rams of both groups would be needed to separate these effects.

Dietary Se supplementation has been associated with higher serum progesterone concentrations during gestation in goats and other ruminants [62,106]. Progesterone, the principal secretory product of the corpus luteum, and later of the placenta, is required for the establishment and maintenance of pregnancy, blastocyst implantation, and the regulation of estrous cycle length [107]. Consistent with these reports, serum progesterone of pregnant ewes was higher in the Se-NP group. This may be related to better luteal or placental function, or to litter size, because two Se-NP ewes carried twins; luteal and placental function were not evaluated in the present study, so these explanations remain speculative. The lower progesterone concentration of the Se-NP ewes at weaning is consistent with an earlier post-partum decline in progesterone preceding the resumption of ovarian cyclicity, but ovarian activity was not monitored, and its physiological significance cannot be established from the present data.

From a metabolic standpoint, supplemental Se-NP may improve lactational performance through several mechanisms, possibly involving enhanced nutrient digestibility, stabilized serum metabolites, and an elevated antioxidant systematic status. Selenium has been reported to support immune function, act as an antioxidant, and enhance nutrient absorption, which may favor mammary gland synthetic capacity and subsequent milk synthesis [108,109,110]. In the present study, Se-NP supplementation was accompanied by an increase in both total milk yield and fat-corrected milk, along with higher concentrations of all milk components. These lactational improvements are supported by earlier investigations showing increased milk, fat, and protein yields in Se-NP supplemented dairy cows and Ossimi ewes [39,42,53,56].

Previous literature reinforces the optimization of growth performance observed in lambs from Se-NP-supplemented ewes, characterized by higher birth weight, weaning weight and ADG than control lambs. For instance, maternal supplementation with 0.3 mg Se-NP/kg DM demonstrates significant improvements in offspring growth performance, including FBW and ADG [42]. This response may be related to the role of Se in the activity of the selenoprotein 5′-deiodinase. This critical enzyme catalyzes the peripheral conversion of prohormone T_4_ into its metabolically active form, T_3_, thereby orchestrating the metabolic signaling pathways required for tissue growth, protein accretion, and cellular energy dynamics [42,74]. Furthermore, the higher ADG may also be related to Se’s pivotal role in bolstering immune competence and enhancing energy utilization efficiency required for skeletal and muscular development [111,112]. Another possible pathway is the placental and lactational transfer of Se from dams to their offspring, which may support neonatal passive immunity and post-natal growth dynamics [30,111]. These phenotypic responses run parallel to earlier investigations in small ruminants [39,60,73]. The faster growth of lambs coincided with higher maternal milk yield, blood Se, and antioxidant capacity in the Se-NP supplemented ewes; however, lamb Se status was not measured and no mediation analysis was performed, so the relative contributions of milk supply, Se transfer, and other maternal effects to lamb growth cannot be determined.

Taken together, supplementation of a low-Se basal diet with 0.3 mg Se-NP/kg DM was associated with improved nutrient digestibility, antioxidant and thyroid status, semen characteristics, a numerically higher conception rate, lactation, and pre-weaning lamb growth, without adverse changes in the hematological variables or body weight measured. These observations do not, however, constitute an evaluation of safety: no toxicological assessment, tissue Se retention, or excretion measurements were performed. Several limitations should be acknowledged. The study was exploratory, with a limited number of animals (6 rams and 15 ewes per group, and 3 rams per breeding group), and no a priori power calculation was performed; rams and ewes were supplemented jointly within breeding groups, so paternal and maternal effects are confounded; the Se-NP product was not characterized beyond the manufacturer’s specifications; the basal Se content was calculated rather than analyzed, and dietary Ca and vitamin E were not determined; only one Se source and dose were tested, and Se-NP was compared with an unsupplemented low-Se control rather than with other Se forms. Future studies should therefore use larger, adequately powered designs with repeated-measures analyses, compare Se-NP with inorganic and organic Se sources at several doses and on basal diets of known (analyzed) Se content, characterize the nanoparticles and their stability in the feed, and directly assess Se absorption, tissue retention, excretion, and safety. Mapping the excretion of nano-mineral residues and their fate in the soil–plant–animal system would also be relevant to the environmental sustainability of this approach.

## 5. Conclusions

Under the conditions of this experiment, supplementation of a low-Se basal diet with 0.3 mg Se-NP/kg DM was associated with improved nutrient digestibility, selected hematological and antioxidant variables, thyroid hormone status, semen characteristics, and serum testosterone in rams, a numerically higher conception rate in the breeding groups, milk production and composition in ewes, and pre-weaning growth of lambs. However, the small number of animals, the group-based reproductive design in which ram and ewe supplementation could not be separated, the low Se content of the basal diet, and the lack of direct measurements of nanoparticle metabolism and toxicity limit the generalization of these findings. Further studies using adequately powered, repeated-measures designs, multiple Se sources and doses, and direct assessment of Se metabolism, safety, and economic returns are warranted before Se-NP supplementation can be recommended for practical use.

## Figures and Tables

**Table 1:** Ingredients and chemical composition of the basal diets.

Items	Rams	Ewes
Ingredients (g/kg DM)		
Corn grain	250	270
Wheat bran	300	250
Soybean meal	120	170
Sugar beet pulp	-	130
Molasses	100	-
Wheat Straw	200	150
Limestone	20	20
Sodium Chloride	10	10
Chemical composition (g/kg DM)		
DM	876.0	892.3
OM	910.1	919.1
CP	144.0	164.6
EE	29.2	29.7
CF	129.9	140.6
ADF	183.4	204.5
NDF	344.0	389.3
NFE	607.0	584.2
Ash	89.9	80.9
Se ^a^, mg/kg DM	0.014	0.005

DM = dry matter; OM = organic matter; CP = crude protein; EE = ether extract; CF = crude fiber; ADF = acid detergent fiber; NDF = neutral detergent fiber; NFE = nitrogen-free extract; Se = selenium. ^a^ Basal Se content was calculated from the ingredient composition using the Se contents of feedstuffs tabulated by the NRC [44]; The supplemented diets contained an additional 0.3 mg Se/kg DM as Se nanoparticles, Se-NP (total = 0.314 and 0.305 mg Se/kg DM for rams and ewes, respectively).

**Table 2:** Body weight and feed intake of Ossimi rams as affected by dietary selenium nanoparticles (Se-NP) supplementation.

Items	Treatments		SEM	*p*-Value
	CON	Se-NP		
IBW, kg	49.25	49.83	2.15	0.7920
FBW, kg	58.27	61.03	2.31	0.2590
DMI, kg/day	1.51	1.56		
DCPI, g/head/day	147.60	163.30		
TDNI, g/head/day	980.30	1040.00		

DMI, DCPI, TDNI were recorded per pen and are presented descriptively; CON = Control; Se-NP = Selenium nanoparticles (0.3 mg/kg DM); SEM = standard error of the mean; IBW = initial body weight; FBW = final body weight; DMI = dry matter intake; DCPI = digestible crude protein intake; TDNI = total digestible nutrient intake.

**Table 3:** Nutrient digestibility and nutritive values of experimental rations in Ossimi rams as affected by dietary selenium nanoparticles (Se-NP) supplementation.

Items	Treatments		SEM	*p*-Value
	CON	Se-NP		
Nutrient digestibility (%)				
DM	68.78 ^b^	78.98 ^a^	0.53	<0.0001
OM	70.70 ^b^	79.81 ^a^	0.41	<0.0001
CP	67.13 ^b^	74.22 ^a^	0.55	0.0002
EE	69.43 ^b^	76.13 ^a^	0.50	0.0002
CF	59.96 ^b^	63.96 ^a^	0.47	0.0011
ADF	43.26 ^b^	44.35 ^a^	0.28	0.0172
NDF	53.89 ^b^	54.54 ^a^	0.22	0.0427
Nutritive values (%)				
DCP	9.77 ^b^	10.87 ^a^	0.03	<0.0001
TDN	64.83 ^b^	68.81 ^a^	0.37	<0.0001

^a, b^ Means within a row with different letters differ significantly (*p* < 0.05); CON = Control; Se-NP = Selenium nanoparticles (0.3 mg/kg DM); SEM = standard error of the mean; DM = dry matter; OM = organic matter; CP = crude protein; EE = ether extract; CF = crude fiber; ADF = acid detergent fiber; NDF = neutral detergent fiber; DCP = digestible crude protein; TDN = total digestible nutrients. Estimated ME (Mcal/kg DM) = 2.34 (CON) and 2.49 (Se-NP).

**Table 4:** Hematological parameters and leukocyte profiles of Ossimi rams as affected by dietary selenium nanoparticles (Se-NP) supplementation.

Items	Treatments		SEM	*p*-Value		
	CON	Se-NP		Treatment	Time	T × T
RBC, ×10^6^/mm^3^	13.97 ^a^	12.53 ^b^	0.27	0.007	0.002	0.160
WBC, ×10^3^/mm^3^	7.68	7.69	0.04	0.899	0.159	0.889
PCV, %	33.29	33.14	0.47	0.830	0.002	0.721
Hb, g/dL	12.67 ^b^	13.28 ^a^	0.14	0.013	<0.001	0.588
Neutrophils, %	30.28	30.25	0.02	0.445	0.003	0.055
Eosinophils, %	4.08 ^b^	4.11 ^a^	0.004	<0.001	0.571	0.407
Basophils, %	0.54	0.54	0.002	0.108	0.778	0.142
Lymphocytes, %	60.89	60.85	0.03	0.358	0.010	<0.001
Monocytes, %	4.21 ^b^	4.26 ^a^	0.009	0.007	0.189	<0.001

Values are least-squares means for months 1–3, adjusted for the pre-treatment (month 0) value, and pooled SEM; n = 6 rams per group. *p*-values are for the effects of treatment, time, and the treatment × time (T × T) interaction in the linear mixed model. ^a, b^ Means within a row with different superscripts differ (*p* < 0.05). CON = Control; Se-NP = Selenium nanoparticles (0.3 mg/kg DM); SEM = standard error of the mean; RBC = red blood cells; WBC = white blood cells; PCV = packed cell volume; Hb = hemoglobin.

**Table 5:** Serum hormonal profiles, serum selenium concentrations, and antioxidant status of Ossimi rams as affected by dietary selenium nanoparticles (Se-NP) supplementation.

Items	Treatments		SEM	*p*-Value		
	CON	Se-NP		Treatment	Time	T × T
T_3_, nmol/L	2.60 ^b^	2.95 ^a^	0.01	<0.001	<0.001	<0.001
T_4_, nmol/L	78.42 ^a^	76.76 ^b^	0.05	<0.001	<0.001	<0.001
T_3_/T_4_ ratio	0.0332 ^b^	0.0385 ^a^	0.0001	<0.001	<0.001	<0.001
TAC, mmol/L	0.509 ^b^	0.803 ^a^	0.003	<0.001	<0.001	<0.001
Se, μg/mL						
Day 0	0.183	0.184	0.0004	0.473		
Day 90	0.286 ^b^	0.556 ^a^	0.004	<0.001		
Testosterone, ng/mL						
Overall (days 30–90)	4.31 ^b^	8.35 ^a^	0.05	<0.001	<0.001	<0.001
Day 0	3.94	3.93	0.05	0.893		
Day 30	4.05 ^b^	5.85 ^a^	0.03	<0.001		
Day 60	4.21 ^b^	7.72 ^a^	0.07	<0.001		
Day 90	4.67 ^b^	11.49 ^a^	0.18	<0.001		

Overall values are least-squares means for months 1–3 (T_3_, T_4_, T_3_/T_4_, and TAC) or days 30–90 (testosterone); n = 6 rams per group, adjusted for the pre-treatment value, and pooled SEM; *p*-values are for the effects of treatment, time, and the treatment × time (T × T) interaction in the linear mixed model. In the time-specific rows, *p*-values are for the comparison of the groups at that time (day 0: one-way ANOVA; days 30–90: Sidak-adjusted comparison of least-squares means). Serum Se: on day 90 was analyzed by ANCOVA with the day-0 value as covariate (n = 6 rams per group). ^a, b^ Means within a row with different superscripts differ (*p* < 0.05). CON = Control; Se-NP = Selenium nanoparticles (0.3 mg/kg DM); SEM = standard error of the mean; T_3_ = triiodothyronine; T_4_ = thyroxine; TAC = total antioxidant capacity; Se = selenium.

**Table 6:** Scrotal circumference and semen physical characteristics of Ossimi rams as affected by dietary selenium nanoparticles (Se-NP) supplementation.

Items	Treatments		SEM	*p*-Value		
	CON	Se-NP		Treatment	Time	T × T
SC, cm	29.19 ^b^	30.71 ^a^	0.41	0.027	<0.001	0.004
EV, ml	1.07 ^b^	1.33 ^a^	0.01	<0.001	<0.001	0.183
Sperm C, ×10^6^/mL	318.5 ^b^	342.9 ^a^	2.9	<0.001	<0.001	0.522
SM, %	80.70 ^b^	85.51 ^a^	0.49	<0.001	0.007	0.006

Values are least-squares means for days 30–90 (SC) or months 1–3 (semen traits), adjusted for the pre-treatment value, and pooled SEM; n = 6 rams per group. *p*-values are for the effects of treatment, time, and the treatment × time (T × T) interaction in the linear mixed model. ^a, b^ Means within a row with different superscripts differ (*p* < 0.05). CON = Control; Se-NP = Selenium nanoparticles (0.3 mg/kg DM); SEM = standard error of the mean; SC = scrotal circumference; EV = ejaculate volume; Sperm C = sperm cell concentration; SM = sperm progressive motility.

**Table 7:** Body weight and feed intake of Ossimi ewes as affected by dietary selenium nanoparticles (Se-NP) supplementation.

Items	Treatments		SEM	*p*-Value
	CON	Se-NP		
IBW, kg	43.30	43.30	2.27	>0.9999
FBW, kg	43.28	42.95	2.80	0.9068
DMI, kg/day	1.76	1.79		
DCPI g/head/day	150.90	173.00		
TDNI g/head/day	840.20	900.90		

DMI, DCPI, TDNI were recorded per pen and are presented descriptively; CON = Control; Se-NP = Selenium nanoparticles (0.3 mg/kg DM); SEM = standard error of the mean; IBW = initial body weight; FBW = final body weight; DMI = dry matter intake; DCPI = digestible crude protein intake; TDNI = total digestible nutrient intake.

**Table 8:** Nutrient digestibility and nutritive values of experimental rations in Ossimi ewes as affected by dietary selenium nanoparticles (Se-NP) supplementation.

Items	Treatments		SEM	*p*-Value
	CON	Se-NP		
Nutrient digestibility (%)				
DM	73.34 ^b^	79.71 ^a^	0.47	<0.0001
OM	76.04 ^b^	83.12 ^a^	0.21	<0.0001
CP	69.94 ^b^	80.17 ^a^	0.59	<0.0001
EE	64.97 ^b^	71.58 ^a^	1.63	0.0154
CF	63.47 ^b^	67.71 ^a^	0.63	0.0024
ADF	44.03	44.19	0.36	0.6886
NDF	50.55 ^b^	54.43 ^a^	0.57	0.0024
Nutritive values (%)				
DCP	11.51 ^b^	13.19 ^a^	0.09	<0.0001
TDN	64.1 ^b^	68.73 ^a^	0.56	0.0012

^a, b^ Means within a row with different letters differ significantly (*p* < 0.05); CON = Control; Se-NP = Selenium nanoparticles (0.3 mg/kg DM); SEM = standard error of the mean; DM = dry matter; OM = organic matter; CP = crude protein; EE = ether extract; CF = crude fiber; ADF = acid detergent fiber; NDF = neutral detergent fiber; DCP = digestible crude protein; TDN = total digestible nutrients. Estimated ME (Mcal/kg DM) = 2.32 (CON) and 2.48 (Se-NP).

**Table 9:** Hematological parameters and leukocyte profiles of Ossimi ewes as affected by dietary selenium nanoparticles (Se-NP) supplementation.

Items	Treatments		SEM	*p*-Value		
	CON	Se-NP		Treatment	Time	T × T
RBC, ×10^6^/mm^3^	11.48 ^b^	11.75 ^a^	0.05	0.002	<0.001	<0.001
WBC, ×10^3^/mm^3^	10.15	10.22	0.04	0.206	<0.001	0.111
PCV, %	36.05 ^b^	37.53 ^a^	0.08	<0.001	<0.001	<0.001
Hb, g/dL	13.16 ^b^	13.80 ^a^	0.05	<0.001	<0.001	0.636
Neutrophils, %	30.38	30.37	0.01	0.351	0.848	0.927
Eosinophils, %	4.056	4.059	0.002	0.374	0.174	0.820
Basophils, %	0.529	0.528	0.001	0.514	0.312	0.656
Lymphocytes, %	60.82	60.83	0.01	0.379	0.907	0.635
Monocytes, %	4.219	4.210	0.006	0.321	0.376	0.155

Values are least-squares means for mating, pregnancy, and weaning, adjusted for the value at the start of the trial, and pooled SEM; n = 15 ewes per group at the start of the trial and at mating; pregnancy and weaning values include only pregnant and lactating ewes (CON,7–9; Se-NP, 14). *p*-values are for the effects of treatment, time, and the treatment × time (T × T) interaction in the linear mixed model. ^a, b^ Means within a row with different superscripts differ (*p* < 0.05). CON = Control; Se-NP = Selenium nanoparticles (0.3 mg/kg DM); SEM = standard error of the mean; RBC = red blood cells; WBC = white blood cells; PCV = packed cell volume; Hb = hemoglobin.

**Table 10:** Serum hormonal profiles, blood selenium concentrations, and antioxidant status of Ossimi ewes as affected by dietary selenium nanoparticles (Se-NP) supplementation.

Items	Treatments		SEM	*p*-Value		
	CON	Se-NP		Treatment	Time	T × T
T_3_, nmol/L	2.69 ^b^	3.34 ^a^	0.04	<0.001	<0.001	<0.001
T_4_, nmol/L	77.31 ^a^	75.99 ^b^	0.33	<0.008	<0.001	<0.001
T_3_/T_4_ ratio	0.0349 ^b^	0.044 ^a^	0.0005	<0.001	<0.001	<0.001
TAC, mmol/L	0.431 ^b^	0.735 ^a^	0.005	<0.001	<0.001	<0.001
Se, μg/mL						
Day 0	0.194	0.193	0.001	0.485		
Weaning (end of trial)	0.312 ^b^	0.585 ^a^	0.002	<0.001		
Progesterone, ng/mL						
Overall (mating to weaning)	5.87 ^a^	5.25 ^b^	0.03	<0.001	<0.001	<0.001
Start of trial	11.18	11.21	0.10	0.877		
Mating	5.28 ^a^	5.22 ^b^	0.01	0.012		
Pregnancy	7.56 ^b^	8.41 ^a^	0.10	<0.001		
Weaning	4.77 ^a^	2.13 ^b^	0.07	<0.001		

Overall values are least-squares means for mating, pregnancy, and weaning, adjusted for the value at the start of the trial, and pooled SEM; n = 15 ewes per group at the start of the trial and at mating; pregnancy and weaning values include only pregnant and lactating ewes (CON, 7–9; Se-NP, 14). *p*-values are for the effects of treatment, time, and the treatment × time (T × T) interaction in the linear mixed model. In the time-specific rows, *p*-values are for the comparison of the groups at that time (start of trial: one-way ANOVA; other stages: Sidak-adjusted comparison of least-squares means). Serum Se at weaning was analyzed by ANCOVA with the initial value as covariate. ^a, b^ Means within a row with different superscripts differ (*p* < 0.05). CON = Control; Se-NP = Selenium nanoparticles (0.3 mg/kg DM); SEM = standard error of the mean; T_3_ = triiodothyronine; T_4_ = thyroxine; TAC = total antioxidant capacity; Se = selenium.

**Table 11:** Reproductive performance of Ossimi ewes and their offspring traits as affected by dietary selenium nanoparticles (Se-NP) supplementation.

Items	Treatments		*p*-Value
	CON	Se-NP	
Maternal reproductive performance			
Ewes exposed to rams, n	15	15	--
Ewes lambing, n	9	14	0.080
Total lambs born, n	9	16	--
Twin-bearing ewes, n	0	2	0.502
Conception rate (%)	(9/15) 60.00	(14/15) 93.33	0.080
Barrenness rate (%)	(6/15) 40.00	(1/15) 6.67	0.080
Fecundity (%)	(9/15) 60.00	(16/15) 106.67	--
Prolificacy (%)	(9/9) 100.00	(16/14) 114.29	--
Offspring survival traits			
Stillborn lambs, n	1	0	--
Surviving lambs at day 7, n	7	15	--
Live-birth rate (%)	(8/9) 88.89	(16/16) 100	0.360
Stillbirth rate (%)	(1/9) 11.11	(0/16) 0.00	0.360
Lamb mortality up to day 7 (%)	(2/9) 22.22	(1/16) 6.25	0.533
Lamb survival up to day 7 (%)	(7/9) 77.78	(15/16) 93.75	0.533

Values are presented as counts and percentages, with the raw counts used for each proportion in parentheses. *p*-values are from the two-tailed Fisher’s exact test. Risk differences (Se-NP - CON, percentage points, 95% CI, Newcombe hybrid score method): conception rate 33.3 (2.6 to 58.2); barrenness rate −33.3 (−58.2 to −2.6); twin-bearing ewes 14.3 (−17.3 to 39.9); live-birth rate 11.1 (−10.3 to 43.5); stillbirth rate-11.1 (−43.5 to 10.3); lamb mortality up to day 7–16.0 (−48.9 to 11.2); lamb survival up to day 7 16.0 (−11.2 to 48.9). Fecundity and prolificacy are lamb-to-ewe ratios and are presented descriptively. CON = Control; Se-NP = Selenium nanoparticles (0.3 mg/kg DM).

**Table 12:** Milk yield and milk composition of Ossimi ewes as affected by dietary selenium nanoparticles (Se-NP) supplementation.

Items	Treatments		SEM	*p*-Value		
	CON	Se-NP		Treatment	Time	T × T
Milk composition, %						
TS	15.33 ^b^	15.99 ^a^	0.07	<0.001	<0.001	<0.001
Fat	6.20 ^b^	6.33 ^a^	0.02	<0.001	<0.001	<0.001
Protein	4.31 ^b^	4.55 ^a^	0.05	0.002	<0.001	<0.001
Lactose	4.20 ^b^	4.34 ^a^	0.04	0.028	<0.001	<0.001
SNF	9.13 ^b^	9.64 ^a^	0.06	<0.001	<0.001	<0.001
Ash	0.651 ^b^	0.763 ^a^	0.008	<0.001	<0.001	<0.001
Milk production, g/d						
MY	517.3 ^b^	675.0 ^a^	12.7	<0.001	<0.001	0.001
FCM	525.3 ^b^	693.0 ^a^	12.6	<0.001	<0.001	0.003
FY	31.99 ^b^	42.61 ^a^	0.77	<0.001	<0.001	0.033
PY	22.36 ^b^	30.66 ^a^	0.73	<0.001	<0.001	0.058
LY	21.71 ^b^	29.11 ^a^	0.63	<0.001	<0.001	0.020

Values are least-squares means for months 0–3 of lactation and pooled SEM; n = 7 (CON) and 14 (Se-NP) lactating ewes. *p*-values are for the effects of treatment, time, and the treatment × time (T × T) interaction in the linear mixed model. ^a, b^ Means within a row with different superscripts differ (*p* < 0.05). TS = total solids; SNF = solids-not-fat; MY = milk yield; FCM = fat-corrected milk; FY, PY, LY = fat, protein, and lactose yields, CON = Control; Se-NP = Selenium nanoparticles (0.3 mg/kg DM); SEM = standard error of the mean.

**Table 13:** Growth performance of suckling lambs as affected by maternal dietary selenium nanoparticles (Se-NP) supplementation.

Items	Treatments		SEM	*p*-Value
	CON	Se-NP		
Birth weight, kg	3.72 ^b^	4.01 ^a^	0.08	<0.001
Weaning weight, kg	21.53 ^b^	24.59 ^a^	0.09	<0.001
ADG, g/day	237.64 ^b^	273.02 ^a^	0.75	<0.001

Values are observed means; SEM and *p*-values are from a linear mixed model with maternal treatment and birth type as fixed effects and the dam as a random effect (Kenward-Roger: degrees of freedom); n = 9 and 16 lambs at birth and 7 and 15 lambs at weaning in the CON and Se-NP groups, respectively. ^a, b^ Means within a row with different superscripts differ (*p* < 0.05). CON = Control; Se-NP = Selenium nanoparticles (0.3 mg/kg DM); SEM = standard error of the mean; ADG = average daily gain.

## Data Availability

The data supporting the findings of this study are available upon request from the corresponding authors.

## Abbreviations

The following abbreviations are used in this manuscript:

ADF	acid detergent fiber
ADG	average daily gain
AIA	acid-insoluble ash
BW	body weight
CF	crude fiber
CON	control
CP	crude protein
DCP	digestible crude protein
DCPI	digestible crude protein intake
DM	dry matter
DMI	dry matter intake
EE	ether extract
EV	ejaculate volume
FBW	final body weight
FCM	fat-corrected milk
FY	fat yield
GPx	glutathione peroxidase
Hb	hemoglobin
IBW	initial body weight
LY	lactose yield
ME	metabolizable energy
MY	milk yield
NDF	neutral detergent fiber
NFE	nitrogen-free extract
OM	organic matter
PCV	packed cell volume
PY	protein yield
RBC	red blood cells
ROS	reactive oxygen species
SC	scrotal circumference
Se	selenium
Se-NP	selenium nanoparticles
SEM	standard error of the mean
SM	sperm progressive motility
SNF	solids-not-fat
Sperm C	sperm cell concentration
T3	triiodothyronine
T4	thyroxine
TAC	total antioxidant capacity
TDN	total digestible nutrients
TDNI	total digestible nutrient intake
TMR	total mixed ration
TS	total solids
WBC	white blood cells

## References

[1] Abdoli R., Zamani P., Mirhoseini S.Z., Ghavi Hossein-Zadeh N., Nadri S. (2016). A review on prolificacy genes in sheep. Reprod. Domest. Anim..

[2] Scaramuzzi R.J., Downing J.A., Campbell B.K., Cognie Y. (1988). Control of Fertility and Fecundity of Sheep by Means of Hormonal Manipulation. Aust. J. Biol. Sci..

[3] Viñoles C., Meikle A., Martin G.B. (2009). Short-term nutritional treatments grazing legumes or feeding concentrates increase prolificacy in Corriedale ewes. Anim. Reprod. Sci..

[4] Maquivar M.G., Smith S.M., Busboom J.R. (2021). Reproductive management of rams and ram lambs during the pre-breeding season in US sheep farms. Animals.

[5] Cameron A.W.N., Oldham C.M., Fairnie I.J., Keogh E.J., Lindsay D.R. (1988). The number of spermatozoa, the number of ovulations per ewe, and immunization against androstenedione affect fertility and prolificacy of sheep. J. Reprod. Fertil..

[6] Tsakmakidis I.A. (2010). Ram semen evaluation: Development and efficiency of modern techniques. Small Rumin. Res..

[7] Kenyon P.R., Morris S.T., West D.M. (2010). Proportion of rams and the condition of ewe lambs at joining influences their breeding performance. Anim. Prod. Sci..

[8] Neary M. (2014). Reproductive Management of the Ewe Flock and the Ram.

[9] Cameron A., Tilbrook A. (1990). The rate of production of spermatozoa by rams and its consequences for flock fertility. Reproductive Physiology of Merino Sheep.

[10] Petrović M.P., Caro Petrović V., Ružić-Muslić D., Maksimović N., Ilić Z.Z., Milošević B., Stojković J. (2012). Some important factors affecting fertility in sheep. Biotechnol. Anim. Husb..

[11] Suttle N. (2022). Mineral Nutrition of Livestock.

[12] Evans A.C.O., Pierson R.A., Garcia A., McDougall L.M., Hrudka F., Rawlings N.C. (1996). Changes in circulating hormone concentrations, testes histology and testes ultrasonography during sexual maturation in beef bulls. Theriogenology.

[13] Tvrdá E., Kňažická Z., Bárdos L., Massányi P., Lukáč N. (2011). Impact of oxidative stress on male fertility—A review. Acta Vet. Hung..

[14] Arshad M.A., Ebeid H.M., Hassan F.-U. (2021). Revisiting the effects of different dietary sources of selenium on the health and performance of dairy animals: A review. Biol. Trace Elem. Res..

[15] Agarwal A., Baskaran S., Parekh N., Cho C.-L., Henkel R., Vij S., Arafa M., Panner Selvam M.K., Shah R. (2021). Male infertility. Lancet.

[16] Dutta S., Majzoub A., Agarwal A. (2019). Oxidative stress and sperm function: A systematic review on evaluation and management. Arab J. Urol..

[17] Hosnedlova B., Kepinska M., Skalickova S., Fernandez C., Ruttkay-Nedecky B., Peng Q., Baron M., Melcova M., Opatrilova R., Zidkova J. (2018). Nano-selenium and its nanomedicine applications: A critical review. Int. J. Nanomed..

[18] Stefanov R., Chervenkov M., Anev G., Maksimović N., Andreeva M., Ivanova T., Milovanović A. (2018). Effect of supplementation with inorganic and organic selenium on sperm quality and quantity in north-east Bulgarian merino rams. Biotechnol. Anim. Husb..

[19] Munoz-Benitez A.L., Vieyra-Alberto R., Hernandez-Medrano J.H., Covarrubias J.L.P., Hernández-Rojas E.G., del Castillo D.A.C.C., Angeles-Hernandez J.C. (2025). Oral selenium supplementation improves the reproductive performance of rams: A systematic review and meta-analysis. Small Rumin. Res..

[20] Talib Ali A.B., Bomboi G., Floris B. (2009). Does Vitamin E or Vitamin E plus Selenium improve reproductive performance of rams during hot weather?. Ital. J. Anim. Sci..

[21] Lee M.R., Fleming H.R., Cogan T., Hodgson C., Davies D.R. (2019). Assessing the ability of silage lactic acid bacteria to incorporate and transform inorganic selenium within laboratory scale silos. Anim. Feed Sci. Technol..

[22] Pecoraro B.M., Leal D.F., Frias-De-Diego A., Browning M., Odle J., Crisci E. (2022). The health benefits of selenium in food animals: A review. J. Anim. Sci. Biotechnol..

[23] Schiavon M., Nardi S., Dalla Vecchia F., Ertani A. (2020). Selenium biofortification in the 21st century: Status and challenges for healthy human nutrition. Plant Soil.

[24] Zoidis E., Seremelis I., Kontopoulos N., Danezis G.P. (2018). Selenium-dependent antioxidant enzymes: Actions and properties of selenoproteins. Antioxidants.

[25] Adadi P., Barakova N.V., Muravyov K.Y., Krivoshapkina E.F. (2019). Designing selenium functional foods and beverages: A review. Food Res. Int..

[26] D’Amato R., Regni L., Falcinelli B., Mattioli S., Benincasa P., Dal Bosco A., Pacheco P., Proietti P., Troni E., Santi C. (2020). Current knowledge on selenium biofortification to improve the nutraceutical profile of food: A comprehensive review. J. Agric. Food Chem..

[27] Wang Z., Tan Y., Cui X., Chang S., Xiao X., Yan T., Wang H., Hou F. (2019). Effect of different levels of selenium yeast on the antioxidant status, nutrient digestibility, selenium balances and nitrogen metabolism of Tibetan sheep in the Qinghai-Tibetan Plateau. Small Rumin. Res..

[28] Ma F., Wo Y., Li H., Chang M., Wei J., Zhao S., Sun P. (2020). Effect of the source of zinc on the tissue accumulation of zinc and jejunal mucosal zinc transporter expression in Holstein dairy calves. Animals.

[29] Gu X., Gao C.-Q. (2022). New horizons for selenium in animal nutrition and functional foods. Anim. Nutr..

[30] Malyugina S., Skalickova S., Skladanka J., Slama P., Horky P. (2021). Biogenic selenium nanoparticles in animal nutrition: A review. Agriculture.

[31] Abdelnour S.A., Alagawany M., Hashem N.M., Farag M.R., Alghamdi E.S., Hassan F.U., Bilal R.M., Elnesr S.S., Dawood M.A., Nagadi S.A. (2021). Nanominerals: Fabrication methods, benefits and hazards, and their applications in ruminants with special reference to selenium and zinc nanoparticles. Animals.

[32] Khalil W.A., El-Harairy M.A., Zeidan A.E.B., Hassan M.A.E. (2019). Impact of selenium nano-particles in semen extender on bull sperm quality after cryopreservation. Theriogenology.

[33] Patra A., Lalhriatpuii M. (2020). Progress and Prospect of Essential Mineral Nanoparticles in Poultry Nutrition and Feeding—A Review. Biol. Trace Elem. Res..

[34] Yuan S., Zhang Y., Dong P.-Y., Chen Yan Y.-M., Liu J., Zhang B.-Q., Chen M.-M., Zhang S.-E., Zhang X.-F. (2024). A comprehensive review on potential role of selenium, selenoproteins and selenium nanoparticles in male fertility. Heliyon.

[35] Hosseini-Vardanjani S., Rezaei J., Karimi-Dehkordi S., Rouzbehan Y. (2020). Effect of feeding nano-ZnO on performance, rumen fermentation, leukocytes, antioxidant capacity, blood serum enzymes and minerals of ewes. Small Rumin. Res..

[36] Semaida A.I., El-Khashab M.A., Mohamed H., Allak M.A. (2025). Impact of nano zinc on hormonal profile, immunity and body/testicular measurements of Ossimi lambs. Fayoum J. Agric. Res. Dev..

[37] Idowu A., Ojebiyi O., Lateef A., Odunsi A., Ola A., Akinola A. (2024). Applications of nanotechnology in animal nutrition: A review. Nano Plus Sci. Technol. Nanomater..

[38] Sitaresmi P.I., Hudaya M.F., Atmoko B.A., Wulandari W., Ujilestari T. (2024). Production and effects of nanomineral selenium (Nano-Se) feed additive on rumen fermentation, productivity, and reproductive performance of ruminants. J. Adv. Vet. Anim. Res..

[39] Ibrahim E.M., Alrauji Y., Elnesr S.S., Shehab-El-Deen M. (2025). Productive Performance and Some Biochemical Indices of Ossimi Ewes and Their Lambs to Dietary Inclusion of Selenium, Zinc Nanoparticles, or Their Combination. Animals.

[40] Shahin M.A., Khalil W.A., Saadeldin I.M., Swelum A.A., El-Harairy M.A. (2020). Comparison between the Effects of Adding Vitamins, Trace Elements, and Nanoparticles to SHOTOR Extender on the Cryopreservation of Dromedary Camel Epididymal Spermatozoa. Animals.

[41] Shi L.-G., Yang R.-J., Yue W.-B., Xun W.-J., Zhang C.-X., Ren Y.-S., Shi L., Lei F.-L. (2010). Effect of elemental nano-selenium on semen quality, glutathione peroxidase activity, and testis ultrastructure in male Boer goats. Anim. Reprod. Sci..

[42] Ibrahim E.M., Mohamed M.Y. (2025). Productive performance and some biochemical indices of Ossimi ewes and their lambs to dietary selenium supplementation. Egypt. J. Sheep Goats Sci..

[43] Khalil M.M.H., Soltan Y.A., Khadiga G.A., Elmahdy A., Sallam S.M.A., Zommara M.A., Rabee A.E., Khattab I.M. (2023). Comparison of dietary supplementation of sodium selenite and bio-nanostructured selenium on nutrient digestibility, blood metabolites, antioxidant status, milk production, and lamb performance of Barki ewes. Anim. Feed Sci. Technol..

[44] NRC (2007). Nutrient Requirements of Small Ruminants: Sheep, Goats, Cervids, and New World Camelids.

[45] Rockville M., AOAC (2023). Official Methods of Analysis of AOAC International.

[46] Goering H., Van Soest P.J. (1970). Forage Fiber Analysis.

[47] Van Keulen J., Young B. (1977). Evaluation of acid-insoluble ash as a natural marker in ruminant digestibility studies. J. Anim. Sci..

[48] Almquist J., Hale E. An approach to the measurement of sexual behaviour and semen production of dairy bulls. Proceedings of the 3rd International Congress on Animal Reproduction.

[49] Hulet C., Foote W., Blackwell R. (1964). Effects of natural and electrical ejaculation on predicting fertility in the ram. J. Anim. Sci..

[50] Marco-Jiménez F., Puchades S., Gadea J., Vicente J., Viudes-de-Castro M. (2005). Effect of semen collection method on pre-and post-thaw Guirra ram spermatozoa. Theriogenology.

[51] Ashmawy G. (1980). Comparison of three techniques for the estimation of the milk production of small ruminants. Egypt. J. Anim. Prod..

[52] Economides S., Louca A. (1981). The effects of the quantity and quality of feed on the performance of pregnant and lactating goats. Nutr. Syst. Goat Feed..

[53] Liu Y., Zhang J., Bu L., Huo W., Pei C., Liu Q. (2024). Effects of nanoselenium supplementation on lactation performance, nutrient digestion and mammary gland development in dairy cows. Anim. Biotechnol..

[54] Xiao M., Wang Y., Wei M., Peng W., Wang Y., Zhang R., Zheng Y., Ju J., Dong C., Du L. (2024). Effects of nanoselenium on the performance, blood indices, and milk metabolites of dairy cows during the peak lactation period. Front. Vet. Sci..

[55] Kareem O., Saadi A., Almallah O. (2024). Addition of selenium nanoscale to high diets with wheat bran and its effect on milk production and composition in Awassi ewes. Assiut Vet. Med. J..

[56] Zhang Z., Wang C., Du H., Liu Q., Guo G., Huo W., Zhang J., Zhang Y., Pei C., Zhang S. (2020). Effects of sodium selenite and coated sodium selenite on lactation performance, total tract nutrient digestion and rumen fermentation in Holstein dairy cows. Animal.

[57] Cui X., Wang Z., Tan Y., Chang S., Zheng H., Wang H., Yan T., Guru T., Hou F. (2021). Selenium yeast dietary supplement affects rumen bacterial population dynamics and fermentation parameters of tibetan sheep (*Ovis aries*) in alpine meadow. Front. Microbiol..

[58] Soltan Y., Morsy A., Hashem N., Elazab M., Sultan M., Marey H., Lail G.A., El-Desoky N., Hosny N., Mahdy A. (2021). Modified Nano-Montmorillonite and Monensin Modulate In Vitro Ruminal Fermentation, Nutrient Degradability, and Methanogenesis Differently. Animals.

[59] Shi L., Xun W., Yue W., Zhang C., Ren Y., Liu Q., Wang Q., Shi L. (2011). Effect of elemental nano-selenium on feed digestibility, rumen fermentation, and purine derivatives in sheep. Anim. Feed Sci. Technol..

[60] Vajpeyee S., Ramesh J., Karunakaran R., Muralidharan J., Sankar V., Das T. (2024). Effect of different selenium sources on nutrient digestibility, performance and antioxidant status in Mecheri lambs. Indian J. Anim. Sci..

[61] Almeida C.F., Faria M., Carvalho J., Pinho E. (2024). Contribution of nanotechnology to greater efficiency in animal nutrition and production. J. Anim. Physiol. Anim. Nutr..

[62] Shi L., Ren Y., Zhang C., Yue W., Lei F. (2018). Effects of organic selenium (Se-enriched yeast) supplementation in gestation diet on antioxidant status, hormone profile and haemato-biochemical parameters in Taihang Black Goats. Anim. Feed Sci. Technol..

[63] Bednarek D., Kondracki M., Cacedillãkała S. (1996). Investigations into the influence of selenium and vitamin E on red and white blood pictures, on concentrations of several minerals and micro-elements in blood serum, and on immunological parameters in calves. Dtsch. Tierärztl. Wochenschr..

[64] Halawa E., Imbabii T., Farid O., Radwan A., ElSayed A. (2023). Ameliorating effect of selenium nanoparticles and L-carnitine on some haemato-biochemical parameters and oxidative stress status during pregnancy periods in Ossimi ewes. Ann. Agric. Sci. Moshtohor.

[65] Shokrollahi B., Mansouri M., Amanlou H. (2013). The Effect of Enriched Milk with Selenium and Vitamin E on Growth Rate, Hematology, Some Blood Biochemical Factors, and Immunoglobulins of Newborn Goat Kids. Biol. Trace Elem. Res..

[66] Mohri M., Ehsani A., Norouzian M.A., Bami M.H., Seifi H.A. (2011). Parenteral Selenium and Vitamin E Supplementation to Lambs: Hematology, Serum Biochemistry, Performance, and Relationship with Other Trace Elements. Biol. Trace Elem. Res..

[67] Soliman E. (2015). Dose-response of vitamin E and selenium injection on growth performance, physiological and immune responses of Ossimi lambs. Egypt. J. Sheep Goats Sci..

[68] Żarczyńska K., Różańska K., Świerczek O., Tobolski D. (2026). The Role of Selenium in the Antioxidant System of Cattle, Pigs, and Small Ruminants: Implications for Animal Health and Productivity. Animals.

[69] Kaneko J.J., Harvey J.W., Bruss M.L. (2008). Clinical Biochemistry of Domestic Animals.

[70] Ahuja A., Parmar D. (2017). Role of minerals in reproductive health of dairy cattle: A review. Int. J. Livest. Res..

[71] Ademi A., Bernhoft A., Govasmark E., Bytyqi H., Sivertsen T., Singh B.R. (2017). Selenium and other mineral concentrations in feed and sheep’s blood in Kosovo. Transl. Anim. Sci..

[72] Ghorbani A., Moeini M.M., Souri M., Hajarian H., Kachuee R. (2024). Effect of dietary zinc, selenium and their combination on antioxidant parameters in serum and Semen of Sanjabi mature rams. J. Trace Elem. Miner..

[73] Salam A., El-Shamaa I., Metwally A., El Hewaty A., Mahmoud T., Zommara M. (2021). Effect of selenium adminstration on reproductive outcome and biochemical parameters to ewes and their lambs. J. Anim. Poult. Prod..

[74] Li L.-P., Qu L., Li T. (2022). Supplemental dietary Selenohomolanthionine affects growth and rumen bacterial population of Shaanbei white cashmere wether goats. Front. Microbiol..

[75] Zheng Y., Xie T., Li S., Wang W., Wang Y., Cao Z., Yang H. (2022). Effects of Selenium as a Dietary Source on Performance, Inflammation, Cell Damage, and Reproduction of Livestock Induced by Heat Stress: A Review. Front. Immunol..

[76] Kehr S., Malinouski M., Finney L., Vogt S., Labunskyy V.M., Kasaikina M.V., Carlson B.A., Zhou Y., Hatfield D.L., Gladyshev V.N. (2009). X-Ray Fluorescence Microscopy Reveals the Role of Selenium in Spermatogenesis. J. Mol. Biol..

[77] Ahsan U., Kamran Z., Raza I., Ahmad S., Babar W., Riaz M.H., Iqbal Z. (2014). Role of selenium in male reproduction—A review. Anim. Reprod. Sci..

[78] Bano I., Malhi M., Soomro S., Kandhro S., Awais M., Baloch S., Perveen S., Sajjad H. (2018). Effect of dietary selenium supplementation on morphology and antioxidant status in testes of goat. J. Basic Appl. Sci..

[79] Qazi I.H., Angel C., Yang H., Zoidis E., Pan B., Wu Z., Ming Z., Zeng C.-J., Meng Q., Han H. (2019). Role of Selenium and Selenoproteins in Male Reproductive Function: A Review of Past and Present Evidences. Antioxidants.

[80] Martins S.M.M.K., Afonso E.R., Parazzi L.J., Andrade A.F.C.D., Leal D.F., Gameiro A.H., Moretti A.D.S.A., Arruda R.P.D. (2018). Organic selenium supplementation is cost-effective for increasing the number of seminal doses produced by sexually mature boars. Rev. Bras. Zootec..

[81] Gabryszuk M., Klewiec J. (2002). Effect of injecting 2- and 3-year-old ewes with selenium and selenium–vitamin E on reproduction and rearing of lambs. Small Rumin. Res..

[82] Makary S., Abdo M., Fekry E. (2018). Oxidative stress burden inhibits spermatogenesis in adult male rats: Testosterone protective effect. Can. J. Physiol. Pharmacol..

[83] Lukusa K., Lehloenya K. (2017). Selenium supplementation improves testicular characteristics and semen quality of Saanen bucks. Small Rumin. Res..

[84] El-Mokadem M., Taha T., Samak M., Yassen A. (2012). Alleviation of reproductive toxicity of gossypol using selenium supplementation in rams. J. Anim. Sci..

[85] Kefer J.C., Agarwal A., Sabanegh E. (2009). Role of antioxidants in the treatment of male infertility. Int. J. Urol..

[86] Agarwal A., Nallella K.P., Allamaneni S.S., Said T.M. (2004). Role of antioxidants in treatment of male infertility: An overview of the literature. Reprod. Biomed. Online.

[87] Gómez-Fernández J., Gómez-Izquierdo E., Tomás C., Mocé E., de Mercado E. (2013). Is Sperm Freezability Related to the Post-Thaw Lipid Peroxidation and the Formation of Reactive Oxygen Species in Boars?. Reprod. Domest. Anim..

[88] Ezaki O. (1990). The insulin-like effects of selenate in rat adipocytes. J. Biol. Chem..

[89] Stapleton S. (2000). Selenium: An insulin mimetic. Cell. Mol. Life Sci. CMLS.

[90] Fürnsinn C., Englisch R., Ebner K., Nowotny P., Vogl C., Waldhäusl W. (1996). Insulin-like vs. non-insulin-like stimulation of glucose metabolism by vanadium, tungsten, and selenium compounds in rat muscle. Life Sci..

[91] Wu A., Oldfield J., Shull L., Cheeke P. (1979). Specific effect of selenium deficiency on rat sperm. Biol. Reprod..

[92] Wang Y., Chen F., Ye L., Zirkin B., Chen H. (2017). Steroidogenesis in Leydig cells: Effects of aging and environmental factors. Reproduction.

[93] Cao L., Leers-Sucheta S., Azhar S. (2004). Aging alters the functional expression of enzymatic and non-enzymatic anti-oxidant defense systems in testicular rat Leydig cells. J. Steroid Biochem. Mol. Biol..

[94] Abidi P., Leers-Sucheta S., Azhar S. (2004). Suppression of steroidogenesis and activator protein-1 transcription factor activity in rat adrenals by vitamin E deficiency–induced chronic oxidative stress. J. Nutr. Biochem..

[95] Erkekoglu P., Giray B., Rachidi W., Hininger-Favier I., Roussel A.M., Favier A., Hincal F. (2014). Effects of di (2-ethylhexyl) phthalate on testicular oxidant/antioxidant status in selenium-deficient and selenium-supplemented rats. Environ. Toxicol..

[96] Ibrahim E., Mohamed M. (2018). Effect of different dietary selenium sources supplementation on nutrient digestibility, productive performance and some serum biochemical indices in sheep. Egypt. J. Nutr. Feed..

[97] Mahmoud G.B., Abdel-Raheem S.M., Hussein H.A. (2013). Effect of combination of vitamin E and selenium injections on reproductive performance and blood parameters of Ossimi rams. Small Rumin. Res..

[98] Marai I.F.M., El-Darawany A.H.A., Ismail E.S.A.F., Abdel-Hafez M.A.M. (2009). Reproductive and physiological traits of Egyptian Suffolk rams as affected by selenium dietary supplementation and housing heat radiation effects during winter of the sub-tropical environment of Egypt (Short Communication). Arch. Anim. Breed..

[99] Mojapelo M.M., Lehloenya K.C. (2019). Effect of selenium supplementation on attainment of puberty in Saanen male goat kids. Theriogenology.

[100] Mojapelo M.M., van Ryssen J.B.J., Lehloenya K.C. (2021). Selenium supplementation reduces induced stress, enhances semen quality and reproductive hormones in Saanen bucks. Small Rumin. Res..

[101] Badgar K., Prokisch J. (2020). The Effects of Selenium Nanoparticles (SeNPs) on Ruminant. Proc. Mong. Acad. Sci..

[102] Neal E.S., Hofstee P., Askew M.R., Kent N.L., Bartho L.A., Perkins A.V., Cuffe J.S.M. (2021). Maternal selenium deficiency in mice promotes sex-specific changes to urine flow and renal expression of mitochondrial proteins in adult offspring. Physiol. Rep..

[103] Elmoghazy M., Ashmawy T., Akl F. (2025). Effect of Supplementation Different Forms of Selenium on Reproductive Performance of Zaraibi Goats. Damietta J. Agric. Sci..

[104] Ali M., El-Sheikh H.A., Ahmed B., Nayel U. (2023). Efficiency of nano zinc supplementation on growth performance, digestibility, rumen parameters, blood biochemistry and immunity status of Barki sheep. Menoufia J. Anim. Poult. Fish. Prod..

[105] Koyuncu M., Yerlikaya H. (2007). *Short Communication* Effect of selenium-vitamin E injections of ewes on reproduction and growth of their lambs. S. Afr. J. Anim. Sci..

[106] Farahavar A., Rostami Z., Alipour D., Ahmadi A. (2020). The effect of pre-breeding vitamin E and selenium injection on reproductive performance, antioxidant status, and progesterone concentration in estrus-synchronized Mehraban ewes. Trop. Anim. Health Prod..

[107] Weems Y.S., Kim L., Tsuda V., Yin C., Weems C.W. (2007). What regulates placental steroidogenesis in 90-day pregnant ewes?. Prostaglandins Other Lipid Mediat..

[108] Tian X., Wang X., Li J., Luo Q., Ban C., Lu Q. (2022). The effects of selenium on rumen fermentation parameters and microbial metagenome in goats. Fermentation.

[109] Silveira R.M.F., Silva B.E.B.E., de Vasconcelos A.M., Façanha D.A.E., Martins T.P., Rogério M.C.P., Ferreira J. (2021). Does organic selenium supplement affect the thermoregulatory responses of dairy goats?. Biol. Rhythm Res..

[110] Rabee A.E., Khalil M.M., Khadiga G.A., Elmahdy A., Sabra E.A., Zommara M.A., Khattab I.M. (2023). Response of rumen fermentation and microbiota to dietary supplementation of sodium selenite and bio-nanostructured selenium in lactating Barki sheep. BMC Vet. Res..

[111] Chen J., Zhang F., Guan W., Song H., Tian M., Cheng L., Shi K., Song J., Chen F., Zhang S. (2019). Increasing selenium supply for heat-stressed or actively cooled sows improves piglet preweaning survival, colostrum and milk composition, as well as maternal selenium, antioxidant status and immunoglobulin transfer. J. Trace Elem. Med. Biol..

[112] Wahyono T., Wahyuningsih R., Setiyawan A., Pratiwi D., Kurniawan T., Hariyadi S., Sholikin M., Jayanegara A., Triyannanto E., Febrisiantosa A. (2023). Effect of dietary selenium supplementation (organic and inorganic) on carcass characteristics and meat quality of ruminants: A meta-analysis. J. Anim. Feed Sci..

